# Converging and emerging threats to health security

**DOI:** 10.1007/s10669-017-9667-0

**Published:** 2017-11-27

**Authors:** C. Raina MacIntyre, Thomas Edward Engells, Matthew Scotch, David James Heslop, Abba B. Gumel, George Poste, Xin Chen, Wesley Herche, Kathleen Steinhöfel, Samsung Lim, Alex Broom

**Affiliations:** 1 School of Public Health and Community Medicine, UNSW Medicine, University of New South Wales, Sydney, NSW 2052 Australia; 2 College of Public Service and Community Solutions and College of Health Solutions, Arizona State University, Tempe, AZ 85287 USA; 3 University of Texas Medical Branch at Galveston, Galveston, TX 77555 USA; 4 Department of Biomedical Informatics, College of Health Solutions, Arizona State University, Phoenix, AZ 85004 USA; 5 School of Public Health and Community Medicine, UNSW Medicine, University of New South Wales, Room 214, Level 2 Samuels Building, Sydney, NSW 2052 Australia; 6 School of Mathematical and Statistical Sciences, Arizona State University, Tempe, AZ 85287 USA; 7 Complex Adaptive Systems Initiative, Arizona State University, Scottsdale, AZ 85257 USA; 8 Office of Knowledge Enterprise Development, Arizona State University, Tempe, AZ 85287 USA; 9 Informatics Department, Natural and Mathematical Sciences, King’s College London, London, WC2R 2LS UK; 10 School of Civil and Environmental Engineering, Faculty of Engineering, University of New South Wales, Sydney, NSW 2052 Australia; 11 School of Social Sciences, Faculty of Arts and Social Sciences, University of New South Wales, Sydney, NSW 2052 Australia

**Keywords:** Biosecurity, Cybersecurity, Terrorism, Disasters, Health intelligence

## Abstract

Advances in biological sciences have outpaced regulatory and legal frameworks for biosecurity. Simultaneously, there has been a convergence of scientific disciplines such as synthetic biology, data science, advanced computing and many other technologies, which all have applications in health. For example, advances in cybercrime methods have created ransomware attacks on hospitals, which can cripple health systems and threaten human life. New kinds of biological weapons which fall outside of traditional Cold War era thinking can be created synthetically using genetic code. These convergent trajectories are dramatically expanding the repertoire of methods which can be used for benefit or harm. We describe a new risk landscape for which there are few precedents, and where regulation and mitigation are a challenge. Rapidly evolving patterns of technology convergence and proliferation of dual-use risks expose inadequate societal preparedness. We outline examples in the areas of biological weapons, antimicrobial resistance, laboratory security and cybersecurity in health care. New challenges in health security such as precision harm in medicine can no longer be addressed within the isolated vertical silo of health, but require cross-disciplinary solutions from other fields. Nor can they cannot be managed effectively by individual countries. We outline the case for new cross-disciplinary approaches in risk analysis to an altered risk landscape.

Advances in biological sciences have occurred at a faster rate than changes in regulatory and legal frameworks for biosecurity. An equally profound change is the convergence of concepts and methods from scientific disciplines that were previously distinct and siloed. Innovations in genomics, synthetic biology, big data, computing science and many other technologies have applications in health and medicine. Equally important, these convergent trajectories are dramatically expanding the repertoire of dual-use technologies—those which can be harnessed for benefit or harm to humanity. Collectively, these trends are shaping a new landscape for systematic risk analysis, for which there are few precedents or analytical tools. These technologies are increasingly available to hostile states and non-state terrorist groups. The rapidly evolving landscape of dual-use risks illustrates how societal preparedness for new challenges in health security can no longer be addressed within isolated vertical siloes. Nor can they cannot be managed effectively by individual countries. Well-recognised threats in the areas of biological weapons, surveillance for bioterrorism, laboratory security and antimicrobial resistance are discussed. We also highlight emerging new areas of threat in the intersection of health security and cybersecurity and enabling of precision harm through big data in medicine. Against this backdrop, we outline below the need for new governance and risk analysis approaches that are global and cross-disciplinary.

## New biological weapons of mass destruction

It has been possible since 2002 to create synthetic viruses in a laboratory, when researchers at the State University of New York at Stony Brook published the synthesis of the poliovirus in *Science* (Cello et al. 2002). There are now over 140 private companies in synthetic biology (Global Engage 2017), which are self-regulated with voluntary codes of conduct (Samuel et al. 2009). Whilst prior biosecurity efforts focused on the biological materials themselves and the inherent challenges of securing and accounting for these materials within laboratories, genetic code can now be transmitted rapidly and used to create new or modified infectious pathogens (Kelle 2009). In the specific case of synthetic infectious agents, the unique characteristic of transmissibility from person-to-person raises special concerns about health security. A virus manufactured in one location may spread worldwide and has a major population impact, thus requiring a global risk mitigation approach. The global regulation of synthetic biology pertaining to communicable diseases is challenging, with many models proposed (Trump et al. 2017). The TAPIC (Transparency, Accountability, Participation, Integrity and policy Capacity) framework provides a guide to good governance (Trump 2017), but there is no enforceable global governance system in place as yet. The many private synthetic biology companies worldwide remain self-regulated, with voluntary guidelines about reporting of suspicious orders. A case in point is smallpox, for which the genome is fully sequenced and publicly available. In a 2017 report, the WHO (World Health Organization 2017d) concluded that destroying existing stocks of smallpox in the USA and Russia would serve no purpose because the virus can be created using synthetic biology. Whilst it is theoretically possible for smallpox to be synthesised in a laboratory, experts have believed this to be a complex task. However, in 2017, Canadian scientists synthetically created an extinct poxvirus, closely related to smallpox, for $100,000 in a laboratory using mail-ordered genetic sequences (Koblentz 2017), illustrating the very real risk of synthetic smallpox emerging as the cause of a pandemic.

In addition to synthetic biology, viruses and bacteria can be engineered for enhanced pathogenicity. A revolutionary new precision tool for gene editing, clustered regularly interspaced short palindromic repeats (CRISPR Cas9) associated nuclease Cas9, raises concerns about dual-use potential (Ran et al. 2013). Whilst CRISPR Cas9 offers the prospect of cures to major diseases, it also enables the precision design and construction of engineered microorganisms as potential weapons of mass destruction. In 2016, the US Director of National Intelligence rated CRISPR Cas9 as a leading weapon of mass destruction threat (MIT Technology Review 2016). Yet global planning for bioterrorism preparedness is still largely framed by cold war concepts and is largely limited to scenarios involving agents such as smallpox and anthrax in a twentieth-century context. However, contemporary biology presents an expanded threat spectrum with an unlimited array of possible engineered agents that transcend the scope of traditional concepts of biosecurity surveillance, preparedness and counter-measures. The convergence of security threats is illustrated by the use enabling technologies such as the dark web for trade in biological weapons and planning of bioterrorist attacks (MacIntyre 2015).

## Challenges in detecting bioterrorism

A natural epidemic is one which arises in nature, without intervention by humans. Unnatural epidemics are those which are caused by human intervention and may be deliberate or accidental release of either naturally occurring or altered pathogens. Bioterrorism is the deliberate release of such pathogens to cause harm (Venkatesh and Memish 2003). Bioterrorism differs from other forms of terrorism in that a bioweapon is microscopic and invisible, and release of a bioweapon is not always recognisable as a deliberate attack (MacIntyre and Engells 2016). All category A bioterrorism agents except smallpox also occur in nature. For example, anthrax occurs more frequently in nature (such as through humans handling infected animal carcasses) than as a bioterrorism attack (Fong and Alibek 2010).

Planning for bioterrorism is underpinned by the assumption that attacks will be recognised as unnatural, but no public health systems exist to differentiate the aetiology of epidemics. Tools such as the Grunow and Finke (2002) criteria are not well known in public health and have low sensitivity for detecting unnatural epidemics when tested against historical events (Chen et al. 2017; MacIntyre and Engells 2016). Public health agencies do not routinely use such tools and default to the assumption that they all epidemics are natural (MacIntyre 2015). There has been an unprecedented increase in the frequency of serious global epidemic risks such as Ebola, avian influenza, MERS coronavirus and Zika virus in recent years (Sands et al. 2016). This cannot be explained solely by environmental and ecological factors, which have changed at a slower rate than the increase in emerging infections. In a recent analysis (Bui et al. 2017), we documented that the rate of emergence of new strains of influenza virus infecting humans is escalating at an unprecedented rate, thereby increasing the probability of a pandemic. Changes in climate, urbanisation and agricultural practices as well as improved global surveillance may have some role in this phenomenon, but have not changed at the same rapid rate as virus evolution. Whether some of these new outbreaks involve engineered agents is unknown and has not been publicly analysed.

Historically, unnatural epidemics have not always been recognised at the time. For example, the Rajneesh salmonella attack in Oregon in 1984 was not only undetected as a bioterrorist attack, but when a local politician suggested bioterrorism, he was ridiculed by public health officials (MacIntyre 2015; Török et al. 1997). Operation Seaspray, which caused a serratia marcescens outbreak in San Francisco in 1950, was not recognised at the time as an open-air test by the US military, which was later disclosed in 1976 (Wheat et al. 1951). The accidental anthrax release in Sverdlovsk, Soviet Union, 1979, was investigated by US experts, who initially incorrectly believed the Soviet explanation that it was a natural outbreak (The Atlantic 2016). There are many other examples of failure to correctly identify unnatural epidemics (Tucker and Zilinskas 2002).

At a time when genetic engineering and synthetic biology contribute to increased risk of biological attacks, there is a need for new tools and risk analysis methods to rapidly identify unnatural epidemics. Yet the Grunow–Finke criteria despite low sensitivity (MacIntyre and Engells 2016) remain the best available tool. Other tools have been developed but are even less known and used than the Grunow–Finke criteria (Chen et al. 2017). In addition to risk analysis tools, rapid surveillance methods are required to detect unnatural epidemic signals. New methods for data mining of open-source unstructured data such as social media show promise for rapid epidemic intelligence (The Conversation 2016) but have not yet been utilised for biosecurity (Yan et al. 2017).

## Laboratory security

Laboratory safety has been highlighted as a key area of concern, with multiple breaches involving security sensitive pathogens in leading laboratories occurring in recent years (Science 2014). Research staff have the unique privilege of working with Biological Select Agents and Toxins and possibly the broader group of Valuable Biological Materials (World Health Organization 2013), yet insider threat and laboratory accidents are known risks. A related issue is practical impacts of the emerging field of Biorisk Management in which the previously separate disciplines of biosafety and biosecurity are merged into a singular approach of Laboratory Biorisk Management (Cook-Deegan et al. 2005; Salerno and Gaudioso 2015) so as to achieve the mutual goal of keeping these materials safe and secure within those areas designated for use and storage. Several entities are proposing this combined approach to enhance the risk management process and produce safer laboratories and more secure practices for these materials (Association of Public Health Laboratories 2016).

A far too common situation at advanced biomedical research laboratories is a problematic relationship between the researchers and security staff. Those relationships must change to reflect a new reality—one in which the research scientist and the law enforcement official are considered members of the same team striving for a mutual goal. Those working in laboratories should monitor each other to create a rigorous form of professional self-governance (Garrett 2013). The implementation of that change will be strategic, for it changes the focus from the substances (biological materials), which because these are alive can be freely found outside the laboratory and continue to defy accurate long-term measurement, to a focus on the behaviours of those who work with these substances—biomedical research scientists, public health officials, clinical laboratorians and others (Aquino 2016).

Another threat is a new facet to the insider threat—the potential for radicalisation of health care workers and researchers, for violent extremists continue to defy precise categorisation or predictable profiles and to infiltrate research institutions (MacIntyre and Engells 2016). In the current age, we must move past our previous solutions dominated by guns, gates and guards and move to a new age in which enhanced personnel security practices through innovative uses of psychology and organisational dynamics will enhance individual and small group accountability and produce a safer laboratory and community at large. Do-it-yourself biology and biohacker labs fall outside of such systems of governance and are presently self-regulated, but the technology is easily accessible for terrorist groups to establish clandestine labs. No systems exist to detect such laboratories. 

## Antimicrobial resistance

The risk of emerging infections is increasing, whilst our ability to treat infections with antibiotics is decreasing. Antibiotics represent one of the major public health achievements of the twentieth century, which together with vaccines are responsible for the dramatic reduction in morbidity and mortality caused by infections. Whilst antimicrobial resistance is not new, its cascading global impact and threat to national and global security are considerable. Within a decade, antimicrobial resistance, driven by prolific misuse in animals, humans and food production, and limited development of new antimicrobial options, will present a significant threat to humanity in the twenty-first century. A key solution is to judiciously use our remaining antibiotic options, yet even in relatively well-off Organisation for Economic Cooperation and Development (OECD) nations, antibiotic misuse continues virtually unabated in both the human and veterinary sectors (World Health Organization 2017b). The World Health Organization (2017b) and other key stakeholders have indicated the critical need for immediate global antimicrobial optimisation, reduction in unnecessary usage, and the roll-out of stewardship across health and agriculture sectors. For example, there has been a 40% global increase in use of last resort antimicrobials (carbapenems) over the last 10 years, and both low-income countries and high-income countries are using substantially more antibiotics per capita than in previous decades (Review on Antimicrobial Resistance 2016).

As with other infectious diseases threats, a global response to this threat is urgently required, with human movement across national borders, threatening antimicrobial viability, even in countries with active surveillance and control programmes. The vertical management of antibiotic use in the human and animal sectors as well as in agriculture and food production must also be addressed in an integrated way, as the volume of use is much higher in these other sectors. A key strategy, in terms of policy, will be the adoption of the One Health model for AMR supported by WHO (2017a), the EU, and the USA (Centers for Disease Control and Prevention 2017). Whilst policy and regulation have progressed considerably in health care environments, a parallel set of strategies will need to be implemented in farming, agriculture and veterinary medicine, which account for around 80% of antibiotic use (Food and Drug Administration 2010). Thus far, strategies utilised across contexts have largely failed to set national targets to reduce antibiotic use in animal agriculture and nor mandate the collection of antibiotic usage data (Martin et al. 2015). Consumer-led strategies—for example, the marketing of antibiotic free products—are one such strategy that may be used to promote sustainable use of antibiotics in the non-health sector (Doyle et al. 2016). Increasing public awareness of the connections between animal and human health—a cornerstone of the One Health approach—will be central to this strategy.

There is growing recognition of the costs and significance of AMR. Multi-resistant organisms are emerging at much higher rates than seen previously, with urgent attention needed to mitigate a risk which is predicted in one report to be the greatest global burden of disease (Review on Antimicrobial Resistance 2016). One recent estimate indicates that by 2050, infections from resistant bacteria may overtake cancer as the leading cause of death in the world and cost US$100 trillion. This estimate has been questioned and likely an overestimate, but AMR nonetheless causes a significant burden of disease (De Kraker et al. 2016). The world is in urgent need of new strategies in the human, animal, agricultural and food industries. This includes reviewing how we price/value antimicrobials, incentives for new antimicrobial development and judicious use, and restrictions around use across sectors. In addition, serious AMR could be engineered and released as an act of bioterrorism, given the availability of technology such as CRISP Cas9 (MacIntyre and Bui 2017). A longer-term model of population risk (versus immediate individual risk of often minor infection) is required to guide everyday use and mitigate this global threat.

## Governance systems for a threat without borders

Whether a bioterrorist attack, pandemic or infections complicated by AMR, the risk is increasing as outlined above. Infectious diseases do not respect international borders and can spread rapidly around the world. The continued growth in large urban areas, and megacities in particular, in which high population densities represent optimum conditions for spread of infection merits significant attention in biosecurity. This risk is heightened for megacities in developing countries in which serious gaps exist in public health surveillance for early detection of epidemic threats, together with inadequate critical infrastructure and other preparedness resources. Prevention, mitigation and control of these threats, therefore, require efforts at local, national and global levels. Despite the call for a One Health approach (Rabinowitz et al. 2013), there is no suitable system for governing use of antimicrobials across human health, animal health and food production, and often no coordination of efforts across these sectors. Global legal and governance frameworks for pandemics and bioterrorism are critical, but there are gaps in some relevant regulations—the International Health Regulations (IHR) (World Health Organization 2017c), the Biological Weapons Convention (BWC) (United Nations Office for Disarmament Affairs 2016) and the Cartagena Protocol (Convention on Biological Diversity 2012). The IHR provides a framework for epidemic preparedness, but many countries do not have the resources to comply with them, and the IHR has not been fully revised since 2005 (World Health Organization 2008). The BWC was revised in 2016, but widely regarded as unenforceable and inadequate in considering new technologies such as CRISPR Cas9 (Bulletin of the Atomic Scientists 2016). The Cartagena protocol was developed to address regulation of movements of living modified organisms (LMOs) resulting from biotechnology from one country to another, but has focused on ecology and biodiversity and has not been utilised for human biosecurity. The TAPIC framework (Trump 2017) is a good starting point for considering how existing regulations can be improved and enforced and how new ones could be developed globally.

## Risk analysis methods for biosecurity

The key needs in risk analysis for biosecurity are timely surveillance and identification of biosecurity threats, risk analysis of impacts and differentiation of natural versus unnatural outbreaks. Traditional disease surveillance lacks the timeliness required for rapid detection of emerging and re-emerging pathogens. Current public health systems rely on validated data from sources health systems, such as hospital and laboratory data, which are important for analysing trends over time, but do not meet rapid epidemic intelligence needs for early detection of epidemics (Yan et al. 2017). Epidemics, defined by a reproductive number greater than one (MacIntyre and Bui 2017), grow exponentially, so every day of delay in detection could substantially increase the morbidity and mortality burden.

Mathematical models, typically of the form of deterministic systems of nonlinear differential equations, are often used to gain insight into the transmission dynamics and impact of natural and unnatural emerging and re-emerging infectious diseases that threaten health security, such as disease pandemics (Nuno et al. 2007; Nuño et al. 2008; Sharomi et al. 2011) and the deliberate release of agents of bioterrorism, such as anthrax (Brookmeyer and Blades 2003; Mushayabasa 2016; Pantha et al. 2016) and smallpox (Banks and Castillo-Chavez 2003; Del Valle et al. 2005; Kaplan et al. 2003; Meltzer et al. 2001). These models, combined with robust health data analytics, computational and data visualisation techniques and numerical simulations provide a realistic, rapid real-time assessment of threats to public health security. Furthermore, informing these models with data generated from modern diagnostic tools that are capable of detecting asymptomatic cases with high degree of sensitivity and specificity (such as peptide-based immune-signaturing or low-cost paper-based RNA sequencing) (Legutki et al. 2014; Navalkar et al. 2014; Pardee et al. 2016; Stafford et al. 2014), and using knowledge of prior bioterrorism attacks and natural disease outbreaks allow for a realistic proactive prediction of future threats before they are detected by the public health system. These approaches allow for a more proactive disease surveillance for human diseases as well as veterinary surveillance for zoonotic pathogens that can mutate and cause major burden in human populations. Other modelling paradigms, such as agents-based and other data-driven statistical and stochastic modelling approaches (Halloran et al. 2002; Hu et al. 2014; Kaplan et al. 2003; Nuño et al. 2008), are also being used for this purpose. A stochastic approach to risk analysis allows model inputs to exhibit a degree of uncertainty. In contrast to deterministic models, the inputs follow various forms of probability distributions. Risk is computed by sampling these input distributions many times. Therefore, the outcome of a stochastic risk model is a distribution of risk—rather than a single value. The key advantage is that in addition to analysing outcomes, it allows for an analysis of the probability of these outcomes. It also allows for easy scenario analysis and sensitivity analysis. These can all assist decision-makers in taking intervention measures and allocating resources. One such example is a stochastic risk model (Hill et al. 2015) of zoonotic and pandemic influenzas, with a focus on human infection with avian influenza. However, there are few real-time models with applicability in operational public health, with most modelling occurring in academia without real-time applicability for disease control (Heslop et al. 2017). End-users in the public health system do not have much knowledge or nor trust in modelling, and do not use it widely for disease control (Muscatello et al. 2017). Availability of simple, transparent tools that can assist with pressing questions such as surge capacity planning during the influenza season is what stakeholders value (Muscatello et al. 2017).

Although mathematical modelling has enjoyed widespread popularity within the academic public health community in terms of using it to predict epidemics as well as to assess and propose effective containment strategies (Newall et al. 2007), the use of genetic data (despite its huge potential to provide much deeper insight) for predictive purposes has not yet become a mainstream tool in public health practice (Dudas et al. 2017). Virus phylogeography and phylodynamics are methods developed to utilize viral DNA sequences to explore the evolution of pathogens by estimating their ancestry. These methods also show promise for public health surveillance, but are not as well developed or used in public health practice. It has only been somewhat recent that more focus has been on combining descriptive phylogenetic approaches with other modelling methods that attempt to predict epidemics. Phylogenetic and phylogeographic analyses can be combined with methods for epidemic modelling to analyse risk factors and predictors of risk in a geospatial and genetic context.

Geographic information systems (GIS) provide a further platform for public health researchers to take a map of an area and add layers of information regarding demographics, disease prevalence and socio-economic status (Mondini and Chiaravalloti-Neto 2008; Rushton 2003). When overlaying multiple types of information, relationships and correlations can be discovered, adding analytical value beyond traditional descriptive approaches (Doku and Lim 2009). Patient data distributions and timestamps are significant factors in determining the specifics of an epidemic disease. Hence, GIS techniques such as emerging hot spot analysis and grouping analysis (Van Steenwinkel et al. 2011) allow for risk analysis of health-related concerns as correlated to location. For example, emerging hot spot analysis (Wang et al. 2008) can help monitor changes and trends of an epidemic disease, such as identifying locations representing new or intensifying hot spots.

New risk analysis methods are required to flag epidemics for urgent intervention, as illustrated by the catastrophic consequences of inaction with the 2014 Ebola epidemic in West Africa (World Health Organization 2015). We have shown that a simple risk prediction tool can be developed which identifies regions at high risk of severe outcomes of epidemics (Argisiri et al. 2017). Such a tool, which considers disease specific, geographic, political, social, situational and contextual factors, could be used to prioritise epidemic response in situations of limited resources and reduce the impact of serious events.

Finally, the vast quantities of publicly available, unstructured data such as news feeds, social media and other public information offer potential for rapid epidemic intelligence and early detection, but are not yet accepted in public health (Yan et al. 2017). Google, Twitter and other sources have shown early promise for rapid disease detection by using algorithms and natural language processing to detect signals for epidemics, but are still eschewed by traditional public health systems (Schmidt 2012). Rapid epidemic intelligence tools based on social media and news feeds could supplement traditional health system based, validated surveillance systems by providing more timely signals for epidemics of concern (Yan et al. 2017). We have shown that the Ebola epidemic of 2014 could have been detected months earlier than it was using a novel Twitter-based tool (Yan et al. 2017).

In addition, risk analysis frameworks play an important role in biosecurity decision-making and policy. Using the approach of multiple criteria based upon emerging biotechnologies such as synthetic biology, traditional risk assessment such as health and environmental data, and other characteristics such as the uncertainty, reversibility, manageability of risk and levels of public concern (Cummings and Kuzma 2017; Trump et al. 2017), can be integrated to provide evidence-based information to government, academia and non-governmental organisations. Such risk analysis methods could assist decision-makers to rank policy priorities and to improve the governance of biosecurity.

Whilst new risk analysis methods and tools are continually developed, many of the established methods for risk analysis of emerging infections are used separately and in different contexts. With increasing biothreats in society, these methods could be better integrated to add value improve the capacity to predict and mitigate risk.

## Biosecurity and cybertechnology

Biosecurity is affected by cybersecurity concerns, but planning for biosecurity often fails to consider critical dependencies with information technology and computing. For example, mitigation and prevention of bioterrorism require surveillance for trade in bioweapons. The dark web offers terrorists a platform for trade in weapons, including bioweapons. Numerous dark web marketplaces such as Silk Road and Alphabay have been shut down by US law enforcement in recent years, but new ones continue to emerge (Business Insider Australia 2017). Whilst such market places are better known for trading in drugs and weapons, in 2015 a New York University student was arrested for attempting to purchase a category B bioterrorism agent, ricin, on the dark web (New York Post 2016). This highlights the need for integration of cybertechnology as a tool in prevention and surveillance for bioterrorism. Surveillance for planned bioterrorism is not as well advanced in this realm as it is for traditional forms of terrorism. Law enforcement agencies employ surveillance of the dark web and social media to detect chatter about planned terrorism, but the focus has been far less on bioterrorism. For example, surveillance for purchase of genetic code to create dangerous viruses failed to pick up the Canadian scientists who created an extinct poxvirus in the laboratory using mail-order DNA (Science 2017). The world first knew about this experiment only when the scientists announced it. Whilst this group was legitimate and not engaged in bioterrorism, the same methods for procurement which they used could well be used by terrorist groups. Therefore, surveillance is required to detect such activity and prevent bioterror attacks.

## Health security and cybersecurity

It was reported in 2017 that the unique health records of all Australians are available for sale on the dark web, a fact uncovered by a reporter, not by law enforcement or government, exposing authorities as completely unprepared (Bickers et al. 2017). Cyberattacks and cybertheft affect all facets of society, from banking and health to critical infrastructure (The Age 2017). In many cases, the outcome of a cyberattack is loss of money or assets. However, if critical infrastructure or health systems are attacked, human lives may be lost. For example, if the power grid of a city is compromised, this has many flow-on effects on health systems, such as loss of functioning of critical equipment in intensive care units or operating theatres, or even for home interventions that rely on power such as nebulisers or oxygen (Lee et al. 2016). Hospitals rely on generators as backup, but these can fail in the event of a disaster (Parson 2002; Sifferlin 2012). The escalation of ransomware attacks on hospitals, which are poorly prepared and easy targets, can bring whole health systems to standstill, as seen within the UK NHS and US hospitals (Deane-McKenna 2017; Gillett 2017; Landi 2017). The goal of most health systems is to achieve paperless operations, to which the electronic patient medical record is central. Yet the zeal for electronic health (E-health) has progressed with very little consideration of cybersecurity, leaving hospitals vulnerable to ransomware attacks (CBSNews 2016).

The aspiration of health systems towards the E-health record is driven by the desire to improve care, protect patient safety and reduce medical errors (Australian Digital Health Agency 2016). The E-health record (EHR) has also been embraced by researchers as a means to efficient health research through data linkage of large administrative databases (Powell and Buchan 2005). For example, data linkage was used to show that CT scans are associated with childhood cancer (Mathews et al. 2013). Whilst big data allows new ways of conducting medical research, the risk of hacking of the EHR has not been adequately mitigated in health care. Education and training in hospital and health management does not routinely offer courses in cybersecurity, leaving health planners and hospital managers unaware of the risk and underprepared. Participants in the US federal Medicare EHR Incentive Program have to attest that they have a fundamental cyber security programme and have adopted certified EHR technology (The Office of the National Coordinator for Health Information Technology 2015). Hospitals attesting Meaningful Use are legally bound to meet specific standards, including protection of the HER, and can be audited. However, even this system has not protected US hospitals from cyberattacks (CBSNews 2016), and many countries do not have any such safeguards.

## A new era of precision harm

**Fig. 1:**
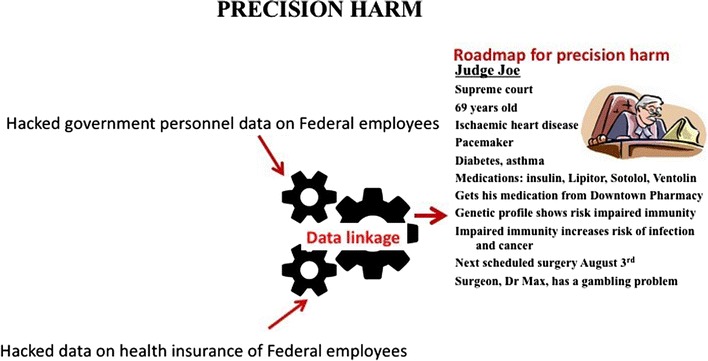
Hypothetical illustration of the use of technology to create precision harm

Precision medicine has revolutionized medicine, with the ability to tailor treatments for individuals by combining detailed medical, genetic and other patient information. This, however, also leads to the possibility of the same information being used to tailor “precision harm”. It is well recognised that individuals can be targeted by biological weapons (MacIntyre and Engells 2016), but convergence of technology opens new avenues for precision harm of individuals. The catastrophic hacking of the US Office of Personnel Management (OPM) in 2014 (Mukherjee 2017) exposed data on over 20 million US Federal employees. At the same time, Anthem Health, the largest provider of health insurance to these employees, was also hacked (Tuttle 2016). Data linkage would allow the perpetrators to access the sensitive personal medical information of employees, identify their medical vulnerabilities and plan targeted attacks, such as medication tampering or hacking of digital medical devices. The risks posed by hacking digital medical devices such as pacemakers and insulin pumps are also cause for concern (Francies 2017). This includes more extreme scenarios in which individuals with high political profiles or other strategic value could be assassinated by manipulation of their medical devices, tampering with their medication regimen or the design of microbial agents matched to their individual genetic profile. Former US Vice President Dick Cheney had his pacemaker wireless function disabled to mitigate such risk (Peterson 2016). If hostile states, organised crime groups or terrorist gain digital medical information on defence or security professionals, government officials or judges, it may become a more attractive option to more obvious methods for causing harm. Figure 1 illustrates the potential for precision harm targeting high profile individuals, enabled by convergence of technologies. In the example provided, a federal judge could be targeted in several different ways, including biological weapons, hacking of digital medical devices, medication tampering, interference with scheduled medical procedures or use of toxins or immune modulators, once a roadmap for precision harm is created. This example could apply to linkage of data from the OPM and Anthem health hacks to create personalised medical profile for federal employees. These examples illustrate the convergence of cybersecurity and health security and the need for more integrated approaches to prevention and mitigation of emerging risks in health care.

## New ways to address risk

In summary, we face rapid advances in science and technology, a corresponding escalation of risk to biosecurity, and convergence of multiple security threats which have traditionally been addressed separately. The changing landscape in biothreats and convergence with other areas of security can no longer be addressed in the traditional narrow, health-centric way. The solution requires a multidisciplinary, global approach to security, whilst meeting local government regulatory requirements. We need new methods to prevent, identify and mitigate threats to biosecurity, which require cooperative thinking across national and professional boundaries. Globally, health, law enforcement, defence and intelligence agencies will need to collaborate and pool their information and expertise. New risk analysis methods and surveillance tools need to be developed, and old methods may need to be used in new ways. This must be addressed in a coordinated global way to ensure risk is minimised.

## References

[CR1] Aquino T (2016). Radicalized health care workers and the risk of Ebola as a bioterror weapon. J Bioterror Biodef.

[CR2] Argisiri S, Chughtai AA, MacIntyre CR (2017) A risk analysis approach to prioritising epidemics—Ebola virus disease in West Africa as a case study. Risk Anal. 10.1111/risa.12876 10.1111/risa.12876 PMC5949606 28810081

[CR3] Association of Public Health Laboratories (2016) Biorisk management for clinical and public health laboratories. https://www.aphl.org/programs/preparedness/Biosafety-and-Biosecurity/Documents/APHL_Biorisk_management_program_guidance_document.pdf. Accessed 6 July 2017

[CR4] Australian Digital Health Agency (2016) Benefits of having a my health record. https://myhealthrecord.gov.au/internet/mhr/publishing.nsf/Content/find-out-benefits. Accessed 25 June 2017

[CR5] Banks HT, Castillo-Chavez C (2003). Bioterrorism: mathematical modeling applications in homeland security.

[CR6] Bickers C, Dunlevy S, Minear T (2017) Hackers are offering to sell the medicare details of Australians on the dark web, government confirms. http://www.news.com.au/technology/online/security/hackers-are-offering-to-sell-the-medicare-details-of-australians-on-the-dark-web-government-confirms/news-story/c475b1cbc963648c191a1eaceba4b12b. Accessed 9 July 2017

[CR7] Brookmeyer R, Blades N (2003). Statistical models and bioterrorism: application to the US anthrax outbreak. J Am Stat Assoc.

[CR8] Bui CM, Chughtai AA, Adam DC, Macintyre CR (2017). An overview of the epidemiology and emergence of influenza A infection in humans over time. Arch Pub Health.

[CR9] Bulletin of the Atomic Scientists (2016) Can the bioweapons convention survive CRISPR? http://thebulletin.org/can-bioweapons-convention-survive-crispr9679. Accessed 15 Sep 2017

[CR10] Business Insider Australia (2017) Authorities just took down AlphaBay, an online black market 10 times bigger than Silk Road. https://www.businessinsider.com.au/alphabay-online-black-market-taken-down-silk-road-2017-7?r=US&IR=T. Accessed 28 Sep 2017

[CR11] CBSNews (2016) Hack on D.C.-area hospital chain reverts them to paper. http://www.cbsnews.com/news/paralyzing-hack-dc-medstar-reverts-them-to-paper/. Accessed 5 June 2017

[CR12] Cello J, Paul AV, Wimmer E (2002). Chemical synthesis of poliovirus cDNA: generation of infectious virus in the absence of natural template. Science.

[CR13] Centers for Disease Control and Prevention (2017) Antibiotic/antimicrobial resistance. https://www.cdc.gov/drugresistance/index.html. Accessed 17 Sep 2017

[CR14] Chen X, Chughtai AA, MacIntyre CR (2017). A systematic review of risk analysis tools for differentiating unnatural from natural epidemics. Mil Med.

[CR15] Convention on Biological Diversity (2012) About the protocol. https://bch.cbd.int/protocol/background/. Accessed 15 Sep 2017

[CR16] Cook-Deegan RM (2005). Issues in biosecurity and biosafety. Science.

[CR17] Cummings CL, Kuzma J (2017). Societal risk evaluation scheme (SRES): scenario-based multi-criteria evaluation of synthetic biology applications. PLoS ONE.

[CR18] De Kraker ME, Stewardson AJ, Harbarth S (2016). Will 10 million people die a year due to antimicrobial resistance by 2050?. PLoS Med.

[CR19] Deane-McKenna C (2017) NHS Ransomware cyber-attack was preventable. https://theconversation.com/nhs-ransomware-cyber-attack-was-preventable-77674. Accessed 25 June 2017

[CR20] Del Valle S, Hethcote H, Hyman JM, Castillo-Chavez C (2005). Effects of behavioral changes in a smallpox attack model. Math Biosci.

[CR21] Doku SA, Lim S (2009) Using GIS to examine the health status of immigrant and indigenous groups in New South Wales, Australia. Paper presented at the GeoComputation, Sydney, Australia, November 30–December 2

[CR22] Doyle M (2016). Enhancing practitioner knowledge about antibiotic resistance: connecting human and animal health. Food Prot Trends.

[CR23] Dudas G (2017). Virus genomes reveal factors that spread and sustained the Ebola epidemic. Nature.

[CR24] Fong IW, Alibek K (2010). Bioterrorism and infectious agents: a new dilemma for the 21st century.

[CR25] Food and Drug Administration (2010) 2009 Summary report on antimicrobials sold or distributed for use in food-producing animals. Accessed 15 Sep 2017

[CR26] Francies R (2017) Medical devices that could put you at security risk. http://www.csoonline.com/article/3192357/internet-of-things/medical-devices-that-could-put-you-at-security-risk.html. Accessed 26 June 2017

[CR27] Garrett L (2013). Biology’s brave new world: the promise and perils of the synbio revolution. Foreign Aff.

[CR28] Gillett F (2017) NHS hospitals hit by major cyber attack as ambulances diverted and operations cancelled. http://www.standard.co.uk/news/uk/nhs-hospitals-hit-by-cyber-attack-creeping-across-england-a3537876.html. Accessed 25 June 2017

[CR29] Global Engage (2017) The big list of synthetic biology companies and investors. http://www.global-engage.com/life-science/list-of-synthetic-biology-companies-and-investors/. Accessed 15 Sep 2017

[CR30] Grunow R, Finke EJ (2002). A procedure for differentiating between the intentional release of biological warfare agents and natural outbreaks of disease: its use in analyzing the tularemia outbreak in Kosovo in 1999 and 2000. Clin Microbiol Infect.

[CR31] Halloran ME, Longini IM, Nizam A, Yang Y (2002). Containing bioterrorist smallpox. Science.

[CR32] Heslop DJ, Chughtai AA, Bui CM, MacIntyre CR (2017) Publicly available software tools for decision-makers during an emergent epidemic-Systematic evaluation of utility and usability. Epidemics. 10.1016/j.epidem.2017.04.002 10.1016/j.epidem.2017.04.002 28576351

[CR33] Hill A (2015). Modelling the species jump: towards assessing the risk of human infection from novel avian influenzas. R Soc Open Sci.

[CR34] Hu S, Barnes S, Golden B (2014) Early detection of bioterrorism: monitoring disease using an agent-based model. In: Simulation conference (WSC), Winter, 2014, IEEE, pp 310–321

[CR35] Kaplan EH, Craft DL, Wein LM (2003). Analyzing bioterror response logistics: the case of smallpox. Math Biosci.

[CR36] Kelle A (2009). Synthetic biology and biosecurity. From low levels of awareness to a comprehensive strategy. EMBO Rep.

[CR37] Koblentz GD (2017). The de novo synthesis of horsepox virus: implications for biosecurity and recommendations for preventing the reemergence of smallpox. Health Security.

[CR38] Landi H (2017) HHS notice: WannaCry Malware continues to impact U.S. healthcare orgs. https://www.healthcare-informatics.com/news-item/cybersecurity/hhs-notice-wannacry-malware-continues-impact-us-healthcare-orgs. Accessed 25 June 2017

[CR39] Lee RM, Assante MJ, Conway T (2016). Analysis of the cyber attack on the Ukrainian power grid.

[CR40] Legutki JB, Zhao Z-G, Greving M, Woodbury N, Johnston SA, Stafford P (2014). Scalable high-density peptide arrays for comprehensive health monitoring. Nat Commun.

[CR41] MacIntyre CR (2015). Biopreparedness in the age of genetically engineered pathogens and open access science: an urgent need for a paradigm shift. Mil Med.

[CR42] MacIntyre CR, Bui CM (2017). Pandemics, public health emergencies and antimicrobial resistance-putting the threat in an epidemiologic and risk analysis context. Arch Public Health.

[CR43] MacIntyre CR, Engells TE (2016). Current biological threats to frontline law enforcement: from the insider threat to diy bio law enforcement executive. Forum.

[CR44] Martin MJ, Thottathil SE, Newman TB (2015). Antibiotics overuse in animal agriculture: a call to action for health care providers.

[CR45] Mathews JD (2013). Cancer risk in 680,000 people exposed to computed tomography scans in childhood or adolescence: data linkage study of 11 million. Aust BMJ.

[CR46] Meltzer MI, Damon I, LeDuc JW, Millar JD (2001). Modeling potential responses to smallpox as a bioterrorist weapon. Emerg Infect Dis.

[CR62] MIT Technology Review (2016) Top U.S. intelligence official calls gene editing a WMD threat. https://www.technologyreview.com/s/600774/top-us-intelligence-official-calls-gene-editing-a-wmd-threat/. Accessed 15 Sep 2017

[CR47] Mondini A, Chiaravalloti-Neto F (2008). Spatial correlation of incidence of dengue with socioeconomic, demographic and environmental variables in a Brazilian city. Sci Total Environ.

[CR48] Mukherjee S (2017) Anthem’s historic 2015 health records breach was likely ordered by a foreign government. http://fortune.com/2017/01/09/anthem-cyber-attack-foreign-government/. Accessed 25 June 2017

[CR49] Muscatello DJ, Chughtai AA, Heywood A, Gardner LM, Heslop DJ, MacIntyre CR (2017). Translation of real-time infectious disease modeling into routine public health practice. Emerg Infect Dis.

[CR50] Mushayabasa S (2016). Dynamics of an Anthrax model with distributed delay acta applicandae mathematicae: an international survey. J Appl Math Math Appl.

[CR51] Navalkar KA, Johnston SA, Woodbury N, Galgiani JN, Magee DM, Chicacz Z, Stafford P (2014). Application of immunosignatures for diagnosis of valley fever. Clin Vaccine Immunol.

[CR52] New York Post (2016) Ex-NYU student gets 16 years in jail for trying to buy ricin. http://nypost.com/2016/03/09/ex-nyu-student-gets-16-years-in-jail-for-trying-to-buy-ricin/. Accessed 28 Sep 2017

[CR53] Newall AT, Beutels P, Wood JG, Edmunds WJ, MacIntyre CR (2007). Cost-effectiveness analyses of human papillomavirus vaccination. Lancet Infect Dis.

[CR54] Nuno M, Chowell G, Gumel A (2007). Assessing the role of basic control measures, antivirals and vaccine in curtailing pandemic influenza: scenarios for the US, UK and the Netherlands. J R Soc Interface.

[CR55] Nuño M, Reichert TA, Chowell G, Gumel AB (2008). Protecting residential care facilities from pandemic influenza. Proc Natl Acad Sci.

[CR56] Pantha B, Day J, Lenhart S (2016). Optimal control applied in an Anthrax epizootic model. J Biol Syst.

[CR57] Pardee K (2016). Rapid, low-cost detection of Zika virus using programmable biomolecular components. Cell.

[CR58] Parson E (2002) 1000-Year flood paralyzes Texas medical center. Electr Constr Maint. http://www.ecmweb.com/contractor/1000-year-flood-paralyzes-texas-medical-center. Accessed 25 June 2017

[CR59] Peterson A (2016) Yes, terrorists could have hacked Dick Cheney’s heart. https://www.washingtonpost.com/news/the-switch/wp/2013/10/21/yes-terrorists-could-have-hacked-dick-cheneys-heart/?utm_term=.cb588b2cd344. Accessed 25 June 2017

[CR60] PLuS ALLIANCE (2017) The PLuS Alliance mobilises to solve problems of global security. http://www.plusalliance.org/press-room/global-security-plus. Accessed 25 June 2017

[CR61] Powell J, Buchan I (2005). Electronic health records should support clinical research. J Med Internet Res.

[CR63] Rabinowitz PM et al. (2013) Toward proof of concept of a one health approach to disease prediction and control. Emerg Infect Dis 19 10.3201/eid1912.130265 PMC3840882 24295136

[CR64] Ran FA, Hsu PD, Wright J, Agarwala V, Scott DA, Zhang F (2013). Genome engineering using the CRISPR-Cas9 system. Nat Protoc.

[CR65] Review on Antimicrobial Resistance (2016) Tackling drug-resistant infections globally: final report and recommendations. Review on antimicrobial resistance. https://amr-review.org/sites/default/files/160518_Final%20paper_with%20cover.pdf. Accessed 8 July 2017

[CR66] Rushton G (2003). Public health, GIS, and spatial analytic tools. Annu Rev Public Health.

[CR67] Salerno RM, Gaudioso J (2015). Laboratory biorisk management: biosafety and biosecurity.

[CR68] Samuel GN, Selgelid MJ, Kerridge I (2009). Managing the unimaginable. EMBO Rep.

[CR69] Sands P, Mundaca-Shah C, Dzau VJ (2016). The neglected dimension of global security—a framework for countering infectious-disease crises. N Engl J Med.

[CR70] Schmidt CW (2012). Trending now: using social media to predict and track disease outbreaks. Environ Health Perspect.

[CR71] Science (2014) Lab incidents lead to safety crackdown at CDC. http://www.sciencemag.org/news/2014/07/lab-incidents-lead-safety-crackdown-cdc. Accessed 20 Sep 2017

[CR72] Science (2017) How Canadian researchers reconstituted an extinct poxvirus for $100,000 using mail-order DNA. http://www.sciencemag.org/news/2017/07/how-canadian-researchers-reconstituted-extinct-poxvirus-100000-using-mail-order-dna. Accessed 28 Sep 2017

[CR73] Sharomi O, Podder C, Gumel A, Mahmud S, Rubinstein E (2011). Modelling the transmission dynamics and control of the novel 2009 swine influenza (H1N1) pandemic. Bull Math Biol.

[CR74] Sifferlin A (2012) Lessons from storm sandy: when hospital generators fail. TIME. http://healthland.time.com/2012/10/30/lessons-from-storm-sandy-when-hospital-generators-fail/. Accessed 25 June 2017

[CR75] Stafford P, Cichacz Z, Woodbury NW, Johnston SA (2014). Immunosignature system for diagnosis of cancer. Proc Natl Acad Sci.

[CR76] The Age (2017) Victoria police cancel hundreds of speeding fines after WannaCry virus attack. http://www.theage.com.au/victoria/victoria-police-cancel-hundreds-of-speeding-tickets-after-wannacry-virus-attack-20170623-gwx7na.html?platform=hootsuite. Accessed 25 June 2017

[CR77] The Atlantic (2016) How DNA evidence confirmed a soviet cover-up of an Anthrax accident. https://www.theatlantic.com/health/archive/2016/11/sverdlovsk-russia-anthrax/508139/. Accessed 28 Sep 2017

[CR78] The Conversation (2016) Social media for tracking disease outbreaks—fad or way of the future? https://theconversation.com/social-media-for-tracking-disease-outbreaks-fad-or-way-of-the-future-66401. Accessed 15 Sep 2017

[CR79] The Office of the National Coordinator for Health Information Technology (2015) Guide to privacy and security of electronic health information

[CR80] Török TJ (1997). A large community outbreak of salmonellosis caused by intentional contamination of restaurant salad bars. JAMA.

[CR81] Trump BD (2017). Synthetic biology regulation and governance: lessons from TAPIC for the United States, European Union, and Singapore. Health Policy.

[CR82] Trump B, Cummings C, Kuzma J, Linkov I (2017) A decision analytic model to guide early‐stage government regulatory action: applications for synthetic biology. Regulation & Governance:e1–e13. 10.1111/rego.12142

[CR83] Tucker JB, Zilinskas RA (2002). The 1971 smallpox epidemic in Aralsk, Kazakhstan, and the Soviet biological warfare program.

[CR84] Tuttle I (2016) Cyberdisaster: how the government compromised our security. http://www.nationalreview.com/article/439869/opm-hack-house-oversight-committee-report. Accessed 25 June 2017

[CR85] United Nations Office for Disarmament Affairs (2016) 8th review conference of the biological weapons convention

[CR86] Van Steenwinkel S, Ribbens S, Ducheyne E, Goossens E, Dewulf J (2011). Assessing biosecurity practices, movements and densities of poultry sites across Belgium, resulting in different farm risk-groups for infectious disease introduction and spread. Prev Vet Med.

[CR87] Venkatesh S, Memish ZA (2003). Bioterrorism–a new challenge for public health. Int J Antimicrob Agents.

[CR88] Wang X, Varady D, Wang Y (2008). Measuring the deconcentration of housing choice voucher program recipients in eight US metropolitan areas using hot spot analysis. Cityscape.

[CR89] Wheat RP, Zuckerman A, Rantz LA (1951). Infection due to chromobacteria; report of 11 cases. AMA Arch Intern Med.

[CR90] World Health Organization (2008). International health regulations (2005).

[CR91] World Health Organization (2013). Biorisk management: laboratory biosecurity guidance (2006).

[CR92] World Health Organization (2015) Report of the Ebola interim assessment panel

[CR93] World Health Organization (2017a) Antimicrobial resistance. http://www.who.int/antimicrobial-resistance/en/. Accessed 15 Sep 2017

[CR94] World Health Organization (2017b) Global action plan on antimicrobial resistance. 2015 ISBN 978:150976

[CR95] World Health Organization (2017c) International health regulations (IHR). http://www.who.int/topics/international_health_regulations/en/. Accessed 15 Sep 2017

[CR96] World Health Organization (2017). WHO advisory committee on variola virus research: report of the eighteenth meeting, 2–3 Nov 2016.

[CR97] Yan SJ, Chughtai AA, MacIntyre CR (2017). Utility and potential of rapid epidemic intelligence from internet-based sources. Int J Infect Dis.

